# Lipodystrophic Laminopathies: From Dunnigan Disease to Progeroid Syndromes

**DOI:** 10.3390/ijms25179324

**Published:** 2024-08-28

**Authors:** Everardo Josué Díaz-López, Sofía Sánchez-Iglesias, Ana I. Castro, Silvia Cobelo-Gómez, Teresa Prado-Moraña, David Araújo-Vilar, Antia Fernandez-Pombo

**Affiliations:** 1 UETeM-Molecular Pathology Group, Department of Psychiatry, Radiology, Public Health, Nursing and Medicine, IDIS-CIMUS, University of Santiago de Compostela, 15706 Santiago de Compostela, Spain; everardodiaz2@gmail.com (E.J.D.-L.); sofia.sanchez@usc.es (S.S.-I.); silviacobelog@gmail.com (S.C.-G.); teresa.prado.morana@sergas.es (T.P.-M.); david.araujo@usc.es (D.A.-V.); 2 Division of Endocrinology and Nutrition, University Clinical Hospital of Santiago de Compostela, 15706 Santiago de Compostela, Spain; anaisabel0121@gmail.com; 3 CIBER Fisiopatología de la Obesidad y la Nutrición (CIBERobn), 28029 Madrid, Spain

**Keywords:** laminopathies, lipodystrophy, Dunnigan disease, FPLD, Hutchinson-Gilford progeria syndrome, mandibuloacral dysplasia, atypical progeroid syndrome, Nestor-Guillermo progeria syndrome, progeria, adipose tissue

## Abstract

Lipodystrophic laminopathies are a group of ultra-rare disorders characterised by the presence of pathogenic variants in the same gene (*LMNA*) and other related genes, along with an impaired adipose tissue pattern and other features that are specific of each of these disorders. The most fascinating traits include their complex genotype-phenotype associations and clinical heterogeneity, ranging from Dunnigan disease, in which the most relevant feature is precisely adipose tissue dysfunction and lipodystrophy, to the other laminopathies affecting adipose tissue, which are also characterised by the presence of signs of premature ageing (Hutchinson Gilford-progeria syndrome, *LMNA*-atypical progeroid syndrome, mandibuloacral dysplasia types A and B, Nestor-Guillermo progeria syndrome, *LMNA*-associated cardiocutaneous progeria). This raises several questions when it comes to understanding how variants in the same gene can lead to similar adipose tissue disturbances and, at the same time, to such heterogeneous phenotypes and variable degrees of metabolic abnormalities. The present review aims to gather the molecular basis of adipose tissue impairment in lipodystrophic laminopathies, their main clinical aspects and recent therapeutic strategies. In addition, it also summarises the key aspects for their differential diagnosis.

## 1. Introduction

Nuclear lamins are crucial nuclear envelope proteins that provide essential structural support and facilitate interactions between extranuclear structures and nucleoplasm components. The A-type nuclear lamins A and C are encoded by the *LMNA* gene [1,2].

Laminopathies are inherited diseases resulting from abnormalities in A-type lamins due to variants in the *LMNA* gene or in other genes involved in prelamin A processing [3,4]. These disorders have diverse clinical manifestations, which may include bone and cardiac abnormalities, lipodystrophy, dermopathy, neuropathy, metabolic abnormalities and premature ageing [5]. In particular, lipodystrophic laminopathies, characterised by selective loss of adipose tissue, represent a distinct subset of these conditions [6].

There are several diseases related to the *LMNA* gene presenting with adipose tissue dysfunction, such as Dunnigan disease or familial partial lipodystrophy type 2 (FPLD2), Hutchinson-Gilford progeria syndrome (HGPS), *LMNA*-associated atypical progeroid syndrome (APS) and mandibuloacral dysplasia type A (MADA) [7]. Among the progeroid syndromes, those associated with *LMNA* variants are particularly significant due to their resemblance to aspects of ageing and range from the mild acceleration observed in MADA to severe acceleration in HGPS [8]. In addition, variants in genes affecting lamin A/C processing and nuclear lamina structure, such as *ZMPSTE24* (linked to mandibuloacral dysplasia type B [MADB]) and *BANF1* (associated with Nestor-Guillermo progeria syndrome [NGPS]), can also result in accelerated ageing syndromes that share adipose tissue impairment [9].

These syndromes underscore the critical role of nuclear envelope proteins in human health and require an interdisciplinary clinical approach to improve diagnosis, monitoring and therapeutic development. This review aims to comprehensively examine lipodystrophic laminopathies, covering the genetic basis of adipose tissue disturbance as a common denominator of these disorders, their clinical manifestations and current therapeutic strategies, in order to improve understanding and guide future research and clinical practice.

## 2. Aetiopathogenesis of Adipose Tissue Impairment in Lipodystrophic Laminopathies

The nuclear lamina is a dense fibrillar network located underneath the inner nuclear membrane. Major components of the nuclear lamina are members of the lamin family of type V intermediate filament proteins, which are important determinants of nuclear and cellular architecture and are significant regulators of stem cell differentiation [10]. The *LMNA* gene codifies for A-type lamins and *LMNB1* and *LMNB2* for lamins B1 and B2, respectively. In this sense, prelamin A is farnesylated, methylated and processed by ZMPSTE24 to form the mature lamina. The correct processing of the A-type lamins is essential for the prevention of laminopathies, which are, as previously mentioned, caused by variants in the *LMNA* gene (such as Dunnigan disease or several premature ageing syndromes), in other genes that influence lamina processing (such as *ZMPSTE24* gene variants in MADB or in restrictive dermopathy) or in genes that influence its proper functioning on chromatin (such as *BANF1* gene variants in NGPS). Thus, *LMNA*-related syndromes are considered to be primary laminopathies and the others secondary laminopathies [10,11,12].

Laminopathies mainly affect mesenchymal tissues (adipose tissue, muscle, bone) [10], although, due to their complex genotype-phenotype associations, not all of them share the same mesenchymal tissue abnormalities. Thus, adipose tissue is affected specifically in the primary lipodystrophic laminopathies Dunnigan disease, HGPS, *LMNA*-atypical progeroid syndrome (APS) and MADA, and in the secondary lipodystrophic laminopathies MADB and NGPS [11]. This raises several questions when it comes to understanding how variants in the same gene can lead to similar adipose tissue disturbances and, at the same time, to such heterogeneous phenotypes.

Thus, the precise mechanisms linking nuclear envelope abnormalities to lipodystrophy remain unknown. However, various theories have been discussed over the years and different pathogenetic mechanisms leading to improper fat distribution in lamin A-linked lipodystrophies have been proposed [10,13,14,15,16,17,18,19,20,21,22,23,24,25]. One such theory is that regulated structural reorganisation of A-type lamins is needed during mesenchymal stem cell differentiation or quiescence. If these rearrangements in the organisation of the lamina are hindered by mutant lamins or their abscence, they could ultimately lead to adipose tissue or muscle impairment [20]. Another theory refers to gene expression and an alteration in transcription factors such as the sterol regulatory element-binding protein 1c (SREBP-1c), which, when sequestered by prelamin A, is not able to properly activate the peroxisome proliferator-activated receptor gamma (PPARG) [18], which is essential in the adipogenesis process. On the other hand, variants in the *LMNA* gene may prevent the correct maturation of prelamin A, generating aberrant electrical charges that alter the interaction with several peptides, such as SREBP-1c itself [26], precluding adipogenesis through the previously mentioned reduction of PPARG activation [27]. Other factors could intervene in this inactivation of PPARG, such as the barrier-to-autointegration factor (BAF) [28] or the retinoblastoma protein (pRb) [29], whose subnuclear localisation and degradation by the proteasome also depend on A-type lamins. pRB promotes adipogenesis, and its phosphorylated form is reduced in lipoatrophic areas in patients with Dunnigan disease [30]. Furthermore, the accumulation of prelamin A alters the nuclear envelope and chromatin organisation, generating oxidative stress, the accumulation of reactive oxygen species [30,31] and a premature ageing phenotype [14,30]. As for the reason why mutant lamin A is unable to complete its maturation, the altered electrical charges previously mentioned were considered responsible by preventing the correct coupling of ZMPSTE24 with prelamin A [32].

Another mechanism proposed to explain the alteration in adipogenesis and premature ageing is the alteration in autophagic flux, which seemed to be reduced [33,34]. Early activation of autophagy in laminopathic adipocyte precursors followed by autophagic flux impairment has also been related to impaired white adipocyte turnover and failure of adipose tissue browning and, therefore, to fat loss and improper accumulation in these syndromes [23]. However, the reason why some areas of the body can be affected and not others, with lamins being ubiquitous proteins, is not completely clear. This could be explained due to the differential expression of different variants of lamins, originated by alternative splicing, establishing different relationships and exerting different functions, depending on the cell type [35].

Nevertheless, the clinical heterogeneity of these disorders also suggests the possible influence of other factors that may even induce epigenetic changes. Thus, one of the hypotheses being raised is that variability in the clinical expressivity of these disorders could be triggered by certain epigenetic marks. Moreover, some studies suggest that the mechanisms responsible for the loss of adipose tissue may be related to certain microRNAs (miRNAs) and histone modifications. In this sense, miRNAs are small, non-coding RNA molecules that play a crucial role in the post-transcriptional regulation of gene expression, usually by mRNA cleavage, mRNA destabilisation or translational repression. They have been shown to be involved in important epigenetic mechanisms such as histone modifications, which are relevant for chromatin formation and the maintenance of the nuclear lamina [21,36,37]. Specifically, the role of dynamic chromatin remodelling on adipogenesis has recently been reported and, more precisely, how pathogenic variants in the *LMNA* gene can modulate certain epigenetic marks such as the anti-adipogenic miRNA miR-335 in adipocyte progenitors, altering the processes of adipocyte differentiation [21].

## 3. Dunnigan Disease

**Figure 1:**
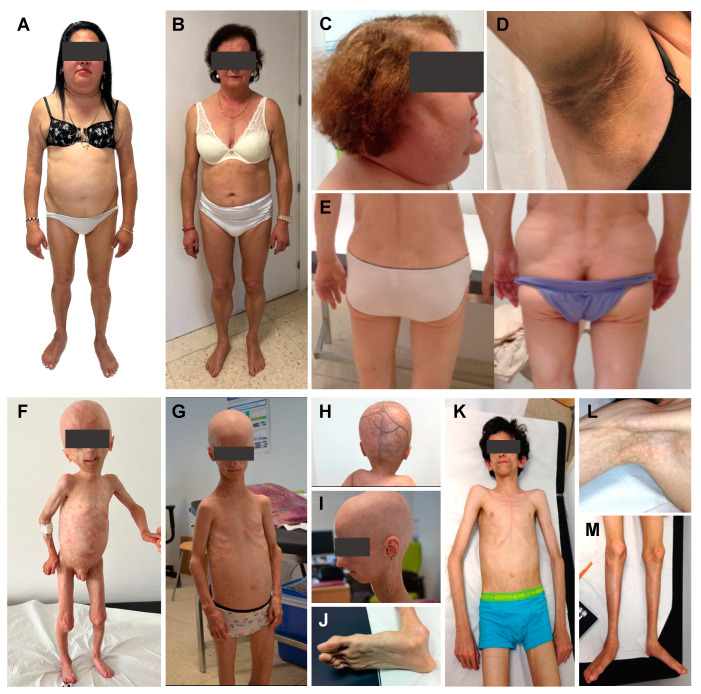
Phenotypic features of patients with lipodystrophic laminopathies. (**A**) 48-year-old woman with Dunnigan disease and classic phenotype due to the p.(Arg482Trp) variant in exon 8 of the *LMNA* gene (FPLD2 #1); (**B**) 62-year-old woman with Dunnigan disease and atypical phenotype due to the p.(Thr528Met) variant in exon 9 of the *LMNA* gene (FPLD2 #2); (**C**) Classic FPLD2 is usually characterised by the accumulation of fat in the face and neck, giving a Cushingoid appearance; (**D**) Signs of insulin resistance such as acanthosis nigricans are common features in FPLD2; (**E**) The presence of subcutaneous lipomas and a concordant phenotype can guide the diagnosis in patients with FPLD2, as is the case of this subject, who developed lipomas within 3 months of follow-up; (**F**) 7-year-old boy with Hutchinson-Gilford progeria syndrome diagnosed at birth due to the 1822G > A, p.(G608S) variant in the *LMNA* gene (HGPS #1), showing the following phenotypical characteristics: generalised lipodystrophy, generalised alopecia, leucomelanodermal macules affecting the entire body, prominent cranial venous tree (**H**), bulging eyes due to the absence of retroorbital fat, small and sharp nose, small ears of normal implantation, dental malposition and absence of teeth, micrognathia and nasal voice. He presented a distended abdomen, with a reducible umbilical hernia and no hepatosplenomegaly. He showed marked veins in the lower limbs. Apparently normal clavicles, nail dysplasia and coxa valga could also be observed. Regarding comorbidities, he presented recurrent infections, bronchial asthma, subclinical hypothyroidism, myopia and, at the age of 5, he was hospitalised due to an occlusive dissection of the left internal carotid artery; (**G**) 16-year-old woman with Hutchinson-Gilford progeria syndrome diagnosed at birth due to the c.1824C > T, p.(G608G) variant in the *LMNA* gene (HGPS #2), showing the following phenotypical characteristics: generalised lipodystrophy, cutaneous sclerosis, generalised alopecia, small and sharp nose, small mouth with dental crowding and micrognathia (**I**). She presented apparently normal clavicles, dysplastic nails, joint stiffness and coxa valga. Regarding comorbidities, she only showed mild aortic insufficiency. However, at the age of 16 she presented an acute myocardial infarction; (**K**) 17-year-old man with *LMNA*-atypical progeroid syndrome diagnosed at 6 months of age due to the heterozygous c.29C > T, p.(Thr10Ile) variant in the *LMNA* gene, showing the following phenotypic characteristics: generalised lipodystrophy affecting palms and soles (**J**,**L**), with thin skin, leucomelanodermal macular lesions (**M**) and a progeroid facies with proptotic eyeballs as a consequence of the probable absence of retro-orbital fat, a pointed nasal pyramid, high-pitched voice and slight crowding of teeth. Hepatomegaly and splenomegaly were palpable. He had joint contractures affecting the upper and lower limbs and metatarsophalangeal stiffness. He presented pseudodislocation of the ankle joint which conditions talus-valgus feet. There was no resorption of the distal phalanges and he had normal nails. He had mild scoliosis, normal clavicles and no mandibular hypoplasia. He presented phlebomegaly in the limbs (**L**). Regarding comorbidities, he was diagnosed with diabetes mellitus, hypertriglyceridaemia, dilated cardiomyopathy with moderate pulmonary hypertension, for which he underwent a heart transplant, and he developed central nervous system lymphoma following the immunosuppressive therapy received. The images included here are of patients from our Lipodystrophy Unit (UETeM reference centre) and have not previously been published elsewhere.

**Figure 2:**
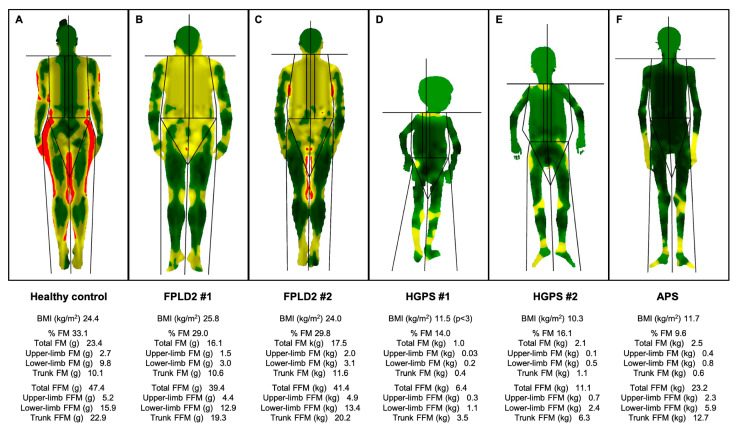
Body composition determined by Dual-Energy X-Ray Absorptiometry of patients with lipodystrophic laminopathies and a non-lipodystrophic subject. Colour mapped, total body composition scans via whole-body Dual-Energy X-Ray Absorptiometry of (**A**) a 42-year-old non-lypodystrophic woman, showing normal fat distribution; (**B**) a 48-year-old woman with classical FPLD2 (FPLD2 #1) and loss of fat in the upper and lower limbs and its accumulation in the face, neck and pubic area; (**C**) a 62-year-old woman with atypical FPLD2 (FPLD2 #2) showing loss of fat in the upper and lower limbs and less accumulation in the face and neck; (**D**) a 7-year-old boy with Hutchinson-Gilford progeria syndrome (HGPS #1), presenting with severe generalised lipodystrophy, including palms and soles as well as muscle atrophy; (**E**) a 16-year-old woman with Hutchinson-Gilford progeria syndrome (HGPS #2), presenting with severe generalised lipodystrophy, including palms and soles, and muscle atrophy; (**F**) a 17-year-old man with *LMNA*-atypical progeroid syndrome, showing generalised lipodystrophy, with preserved adipose tissue in the palms, loss of fat in the soles and muscle mass atrophy. Green represents an area of low level % fat (0–25%), yellow an area of medium level % fat (25–60%), and red an area of high level % fat (60–100%). These are original data from patients of our Lipodystrophy Unit (UETeM reference centre) and have not previously been published elsewhere. FPLD2: familial partial lipodystrophy type 2; HGPS: Hutchinson-Gilford progeria syndrome; APS: *LMNA*-atypical progeroid syndrome; BMI: body mass index; FM: fat mass; FFM: fat-free mass.

Dunnigan disease, also known as FPLD2 (MIM#151660), is characterised by the loss of adipose tissue from the trunk, buttocks and upper and lower limbs mainly from puberty, associated with an accumulation of fat in the face, neck, supraclavicular fossae and labia majora/pubic area [38,39,40,41]. Most of the subjects with this phenotype harbour heterozygous missense variants affecting arginine at codon 482 in exon 8 of the *LMNA* gene. However, variants in other exons have also been reported and are shown to promote atypical FPLD2, with a variable pattern and severity of fat loss [42,43,44,45,46,47,48,49,50,51] (Figure 1 and Figure 2). There is also a tendency towards the formation of subcutaneous lipomas (found in 20% of cases), which can guide the diagnosis in subjects with a concordant phenotype [30] and, along with this reported abnormal pattern of fat distribution, muscular hypertrophy and myalgias are also recurrent features [52,53,54]. Identification of the “Dunnigan sign” (hypertrophy of mons pubis fat surrounded by subcutaneous lipoatrophy) is likewise helpful in diagnosing women with this disorder. Due to their lack of subcutaneous fat, these patients tend to show very prominent peripheral veins [55]. Signs of hyperandrogenism, such as hirsutism, can be seen in affected women [52,56], as well as the presence of signs of insulin resistance, including acrochordons and acanthosis nigricans [57,58,59] (Figure 1). The clinical diagnosis of this disorder is not difficult in women for trained physicians considering that the expression and severity of the previously mentioned phenotype may be markedly dependent on sex, with women being more affected. On the contrary, men typically show a less-apparent lipodystrophy phenotype and a later onset [41,42].

Dunnigan disease can become a serious disorder with potentially lethal associated comorbidities and metabolic complications. Although these comorbidities usually develop after the age of 10, an anticipation phenomenon has been described, with the occurrence of metabolic disturbances at an earlier age across generations [60,61,62]. In this sense, the response of maladaptive adipose tissue acquires a dysfunctional pattern of adipocytokine production. These adipocytokines, synthesised both by adipocytes and by other inflammatory cells, interfere with insulin signalling pathways, generating resistance to its action [63]. Ectopic fat accumulation, mainly in the liver and muscle, also plays a relevant role in the aetiopathogenesis of insulin resistance. This can lead to a variable degree of metabolic complications, including non-ketotic diabetes, with a prevalence ranging from 28% to 51% [58,64,65,66,67,68], or dyslipidaemia, which is fundamentally characterised by high triglyceride levels, also related to acute pancreatitis in some of these subjects [69], and low high-density lipoprotein cholesterol [46,58,61,70,71,72,73]. Hypertension has also been described with a prevalence of up to 41% [46,59,74]. Kidney alterations including proteinuria and renal failure have likewise been reported [75]. Non-alcoholic fatty liver disease and non-alcoholic steatohepatitis, ultimately leading to cirrhosis, are other frequent organ abnormalities due to ectopic lipid storage in the liver [58,76]. An increased prevalence of cardiac manifestations, such as cardiac hypertrophy [56,77], atrioventricular conduction defects, heart failure due to ventricular dilatation [78,79], early atherosclerosis [80,81,82] and arrhythmias [78,79,83], has also been described in this population. Polycystic ovary syndrome, fertility problems and a higher rate of miscarriages and stillbirths can likewise be observed [84,85,86,87].

Furthermore, it should be taken into account that Dunnigan disease, in addition to belonging to the laminopathy family due to the involvement of the *LMNA* gene in its aetiopathogenesis, also belongs to the FPLD group of syndromes, which comprises a total of nine FPLD subtypes and another three unclassified variants of FPLD [88,89,90,91,92,93,94,95,96,97,98,99,100,101,102,103,104,105,106,107,108,109,110]. This set of disorders shares most of the previously mentioned phenotypic characteristics and comorbidities and, therefore, attention should be paid to certain peculiarities of each specific disease that may help to perform a correct differential diagnosis. The main differential characteristics of Dunnigan disease and the other 11 FPLD subtypes can be seen in Table 1.

There is currently no cure for this disorder and fat loss is generally not recovered. However, the morbidity and mortality of this condition could be improved with early intervention and, therefore, its treatment should be fundamentally oriented towards the control of the metabolic complications previously described. Apart from diet, along with physical activity (after an adequate cardiological assessment and avoiding vigorous exercise) and standard treatments mainly aimed at the control of diabetes and dyslipidaemia, several drugs have been specifically tested in this population. Metreleptin, a human recombinant leptin, has been approved by the European Medicines Agency (EMA) for the treatment of FPLD subjects > 12 years of age who have not responded to standard therapies (glycated haemoglobin [HbA1c] > 8% and/or triglycerides > 500 mg/dL) (https://www.ema.europa.eu/en/medicines/human/EPAR/myalepta, accessed on 7 July 2024). However, its response in partial lipodystrophy is considered to be less robust than in generalised forms. In this sense, while metreleptin has been proven to reduce serum triglyceride levels regardless of patients’ leptin levels, the reduction in HbA1c was found to be inconsistent [111,112,113]. It also seems to improve hepatic steatosis [114], although more studies in this field and in this specific population are needed. On the other hand, new drugs focused on hepatic lipid metabolism have also been investigated in recent years with promising results, as is the case of volanesorsen, an antisense inhibitor of apolipoprotein C-III. Its efficacy and safety have been evaluated in a 52-week phase II/III study, showing an 88% decrease in triglycerides and a significant reduction in hepatic fat fraction in 40 patients with FPLD [115]. Less robust results have been observed for gemcabene calcium, a monocalcium salt of a dialkyl ether dicarboxylic acid, the efficacy and safety of which has been evaluated in five women with FPLD (two with the *LMNA* R482Q variant), showing a median change in serum triglycerides of −19.6% [116]. Vupanorsen, an inhibitor of ANGPTL3, has also been studied in a small number of patients with FPLD (two of them with Dunnigan disease) showing a reduction of triglyceride fasting levels of 59.9%, as well as in other lipoproteins [117]. In addition, a phase II study regarding the effectiveness of obeticholic acid in reducing hepatic triglyceride levels in patients with Dunnigan disease and hepatic steatosis has recently been completed (ClinicalTrials. gov Identifier: NCT02430077).

## 4. Hutchinson-Gilford Progeria Syndrome

With an estimated prevalence of one case per 20 million inhabitants (www.progeriaresearch.org; accessed on 7 July 2024), HGPS (#MIM176670) is considered to be one of the most severe laminopathies, originating from the aberrant splicing of the *LMNA* gene and the consequent expression of an abnormal lamin A protein, called progerin [118,119]. Patients are healthy at birth with the phenotype usually becoming evident at 18–24 months of age, including a broad range of clinical features [120,121,122] (Figure 1 and Figure 2): low body weight, with severe generalised lipodystrophy while preserving intraabdominal fat, along with muscular atrophy, growth retardation and short stature, early alopecia with prominent scalp veins, loss of skin elasticity and skin hypo/hyperpigmentation, beaked nose, micrognathia, high-arched palate, mandibular osteolysis, dental crowding, high-pitched voice, sensorineural hearing loss, nail dystrophy, joint stiffness, short club-shaped distal phalanges (acroosteolysis) and osteopenia. Patients with HGPS do not show intellectual impairment [119].

The most significant abnormalities in HGPS, which ultimately lead to death at around the age of 14 [123], are cardiovascular complications (myocardial ischemia, infarction and stroke) [124]. Patients can suffer insulin resistance with some showing elevated levels of serum triglycerides, total cholesterol and low-density lipoprotein cholesterol with reduced levels of high-density lipoprotein cholesterol [122,125]. However, interestingly, metabolic derangements are mild and HGPS patients rarely develop dyslipidaemia or increased C-reactive protein, two characteristics often observed in cardiovascular disease in the general population [124,126]. This leads to the suspicion that the mechanisms leading to the development of these cardiovascular complications in this group of patients differ from the normal ageing population. Thus, beyond atherosclerosis, there is evidence of intimal thickening and an increase in arterial stiffness leading to many measurable changes in the vasculature of these patients [122,127]. They also present vascular walls with a dramatically thickened fibrotic matrix with a depletion of medial smooth muscle cells that is replaced by collagen, with secondary maladaptive vascular remodelling, probably due in part to the extreme sensitivity of these cells to progerin expression [122,127,128,129,130].

Even though there is currently no cure for this disorder, in recent years, a number of therapies have shown promise in preclinical stages for its treatment and, in 2020, lonafarnib became the first (and only) ever U.S. Food and Drug Administration (FDA) and EMA approved treatment for this condition. This drug is a farnesyltransferase inhibitor, which works by inhibiting the processing of prelamin A to progerin [131,132,133], the administration of which has been shown to improve some of the symptoms of the disease (such as rate of weight gain, pulse-wave velocity, carotid artery wall echodensity, skeletal rigidity, cardiovascular stiffness, bone density and sensorineural hearing) and also a decreased mortality rate [132,134,135]. In combination with lonafarnib, the rapamycin analog everolimus is also currently being tested in an ongoing phase I/II clinical trial (ClinicalTrials.gov Identifier: NCT02579044). Likewise, particularly remarkable is the addition of progerinin as a new candidate specifically developed for progeria, the safety, tolerability, pharmacokinetics and pharmacodynamic profile of which is being studied in another phase I clinical trial (ClinicalTrials.gov Identifier: NCT04512963). Furthermore, a potential genetic therapeutic strategy employing antisense peptide-conjugated phosphorodiamidate morpholino oligomers (PPMOs) to block the pathogenic splicing of mutant transcripts has shown a significant reduction of progerin transcripts in the aorta, a 61.6% increase in lifespan and rescue of vascular smooth muscle cell loss in large arteries in transgenic mouse models of HGPS [136]. CRISPR/Cas9 gene editing also seems a promising strategy for the treatment of genetic diseases, including HGPS [137].

## 5. *LMNA*-Atypical Progeroid Syndrome

This syndrome, due to different missense *LMNA* variants (such as P4R, E111K, D136H, E159K, C588R, R349W or T10I) is characterised by a marked phenotypic heterogeneity, with evidence of both partial and generalised lipodystrophy (Figure 2). While the accumulation of farnesylated prelamin A has been demonstrated in some cases, in other cases the pathogenesis of clinical manifestations was not related to this accumulation [138,139,140].

The onset of the phenotype occurs later than in HGPS and MAD, during childhood or early adulthood, [139,141,142] and life expectancy is likewise longer [143]. Patients with APS usually share several progeroid features (Figure 1), including short stature, partial alopecia, early greying of hair, mottled skin pigmentation, sclerodermiform lesions, beaked nose, high-arched palate, micrognatia, abnormal teeth implantation with dental crowding, high-pitched voice, sensorineural hearing impairment, joint stiffness and osteoporosis [138,139,144]. However, although they have overlapping features when compared with HGPS and MAD patients, acroosteolysis and clavicular resorption/hypoplasia are usually absent or mild in this specific disorder [139]. On the other hand, this syndrome is frequently associated with metabolic abnormalities and cardiovascular complications, including valvular disease, rhythm disturbances, coronary artery disease and cardiomyopathy. Proteinuric nephropathy is also present in the majority of cases [139,142,144,145].

In 2018, Hussain I et al. proposed the designation of a distinctive syndrome due to heterozygous *LMNA* p.T10I variants, called generalised lipodystrophy-associated progeroid syndrome, considering the unique and relatively homogeneous clinical features of this disorder in comparison with the previously reported APS. These specific characteristics were early childhood onset of generalised lipodystrophy along with other progeroid features, more severe metabolic complications and a notable need for cardiac transplantation [140]. Another recurrent variant is the previously mentioned R349W, which leads to a multisystem progeroid syndrome with lipodystrophy characterised by a loss of fat in the limbs and face and its accumulation in the dorsocervical region, along with the shared progeroid features described [141,142]. On the other hand, the missense *LMNA* variant p.(Thr528Met) was identified in heterozygosity in subjects with FPLD2, in a compound heterozygous state in subjects with APS and severe partial lipodystrophy and, recently, in homozygosity in subjects with homogeneous APS clinical features with major musculoskeletal involvement [146].

Regarding the availability of specific therapies, it should be noted that metreleptin has been successfully used in some isolated cases for the treatment of associated comorbidities [147].

## 6. Other *LMNA*-Associated Lipodystrophies

### 6.1. LMNA-Associated Generalised Lipodystrophy

Only one case with the p.(Arg582His) variant in exon 11 of the *LMNA* gene in a homozygous fashion and near-total fat loss has been reported. However, adipose tissue was found to be preserved in the retroorbital area, mons pubis and the genital region. This homozygous variant was also associated with an earlier onset of metabolic complications in comparison to heterozygous cases [148]. Another family of four with the p.(Arg582Cys) variant in exon 11 of the *LMNA* gene in the homozygous and heterozygous states has been described. All members showed extremely distinct features including a severe generalised lipodystrophic phenotype and metabolic abnormalities leading to death at a young age [47]. Finally, although the pathogenic p.(Arg571Ser) variant in the *LMNA* gene has been previously associated with cardiomyopathy and neuropathy without lipodystrophy [149,150], in 2017, another two cases harbouring generalised loss of fat were also described. The onset of the phenotype in these cases occurred after birth and in early childhood with both subjects developing severe metabolic abnormalities and dying at an early age [151].

### 6.2. LMNA-Associated Cardiocutaneous Progeria

Only one patient has been reported with this specific syndrome due to the p.(Asp300Gly) variant in the *LMNA* gene developing an unspecified pattern of fat loss around the third decade of life. Cardiac and cutaneous manifestations along with a prematurely aged appearance were also described for this patient. In addition, it should be noted that this disorder may also be related to a greater incidence of malignancy, an unprecedented finding in *LMNA*-linked progeria disorders [152].

## 7. Mandibuloacral Dysplasia

Patients with MAD present with a range of clinical manifestations. Facial features typically include prominent scalp veins, ocular proptosis, beak-like nose, dental crowding and progressive osteolysis affecting the mandible, terminal phalanges and clavicles. Skeletal abnormalities include acroosteolysis, prominent interphalangeal joints and dystrophic nails [153]. Cutaneous manifestations in MAD include thin, wrinkled skin with mottled hyperpigmentation and atrophy in the acral regions, along with visible veins and tendons due to the absence of subcutaneous fat [154]. Although skeletal and cardiac muscles are usually unaffected in most cases of MAD, muscle weakness may occur with specific gene variants [155]. Features of metabolic syndrome such as insulin resistance, glucose intolerance, and hypertriglyceridemia have also been documented among affected individuals [156].

Two distinct patterns of body fat distribution are observed in patients with MAD-associated lipodystrophy. The first pattern (type A) involves loss of fat from the limbs along with normal or increased accumulation of fat in the face, neck and trunk. In contrast, the second pattern (type B) is characterised by a generalised loss of subcutaneous fat. A notable phenotypic difference lies in the specific association of *LMNA* variants with partial lipodystrophy (MADA) and *ZMPSTE24* variants with generalised lipodystrophy (MADB) [157,158].

### 7.1. Mandibuloacral Dysplasia Type A

MADA (#MIM 248370) was first described in 1971 [159] as a rare autosomal recessive disorder resulting from abnormalities in nuclear lamin proteins, specifically lamins A and C [160]. MADA is predominantly caused by homozygous variants in the *LMNA* gene, leading to the accumulation of mutated prelamin A and lamin A/C [161]. Although most cases involve homozygous *LMNA* variants, there are instances of less frequent heterozygous compound conditions [162].

Symptoms typically appear in early childhood, becoming more pronounced during adolescence [153]. The clinical presentation of MADA exhibits significant homogeneity, despite its diverse impact on multiple organ systems among individuals [161]. A proposed classification categorises major clinical features seen in over 75% of patients, including acroosteolysis (100%), lipodystrophy (98%), mandibular hypoplasia (95%), clavicular hypoplasia (93%), growth retardation (79%) and a beaked nose (77%). Manifestations observed in 50–75% of patients include mottled skin pigmentation (72%), prominent cheeks (70%), prominent eyes (65%) and dental crowding (63%). Alopecia is a less common sign, reported in approximately half of the patients [163]. Female MADA patients may also present with lack of breast development and irregular menstrual periods [164].

MADA patients commonly exhibit lipodystrophy, characterised by partial loss of subcutaneous fat in the limbs and excessive fat deposition in the neck and trunk. In addition, variability in neck fat distribution is noted among patients, with some exhibiting normal fat levels while others develop fat accumulation, resembling a buffalo hump [14]. This pattern of fat distribution is reminiscent of that observed in FPLD2, also caused by *LMNA* gene variants [14,158]. In addition to skeletal and adipose tissue abnormalities, MADA is associated with mildly accelerated ageing, generalised joint stiffness and bone abnormalities that progress with age [165,166].

Overall, the comprehensive understanding of MADA requires ongoing research and clinical vigilance to manage its varied manifestations effectively. Current treatment focuses on managing the symptoms and complications associated with the condition, as there are no specifically approved therapies. Management should involve a multidisciplinary approach to address various aspects of the disorder, such as skeletal abnormalities, skin changes and potential cardiac issues.

Various cellular treatments have been developed to enhance the phenotype of cultured MADA cells by reducing accumulated prelamin A. Statins and farnesyl transferase inhibitors (FTIs) inhibit farnesylated prelamin A accumulation. Treatment of MADA cultured cells with statins was effective in the recovery of the chromatin phenotype, which is altered in MADA cells, but only in low passage cultures [161]. Combined treatment with mevinolin and trichostatin A improved the effects observed with mevinolin alone [161]. In addition, MADA fibroblasts subjected to chloroquine treatment showed increased levels of prelamin A, while impairing the autophagic process, suggesting an autophagic mechanism for removal of mutated prelamin A in these cells [8]. Moreover, inhibition of the mTOR pathway by rapamycin triggers lysosomal degradation of farnesylated prelamin A in MADA fibroblasts and rescues markers of cellular senescence as well as chromatin epigenetic mechanisms [167].

On the other hand, as previously described, patients affected by MADA suffer from an osteolytic process due to an excessive amount of released TGF-β2. Neutralising antibodies against TGF-β2, as well as statins or rapamycin, have been shown to block this osteolytic process, indicating potential therapeutic tools for MADA treatment. Everolimus treatment significantly improves the mutant phenotype and its pathogenetic pathways, suggesting it could be a therapeutic approach for MADA [168]. In this context, cytokines and other circulating molecules warrant further investigation as potential therapeutic targets for this syndrome [153].

### 7.2. Mandibuloacral Dysplasia Type B

MADB (#MIM 608612) is a rare premature ageing disorder following an autosomal recessive inheritance pattern [157]. MADB is primarily caused by variants in the *ZMPSTE24* gene, resulting in impaired enzymatic activity and accumulation of farnesylated prelamin A [169].

Variants in the *ZMPSTE24* gene have been associated with progeroid syndromes, including MADB and restrictive dermopathy, as well as a severe metabolic syndrome with abnormal fat accumulation and dilated cardiomyopathy [170]. However, patients with MADB typically present a more generalised loss of subcutaneous fat [171]. The median age at diagnosis is significantly younger (approximately 4 months) than subjects with MADA (approximately 4 years), suggesting a more severe phenotype [157].

The proposed diagnostic criteria for MADB require the presence of four or more major clinical criteria or the presence of three or more major clinical criteria and two or more minor clinical criteria. Major criteria include: short stature (height less than 2 standard deviations), clavicular hypoplasia, delayed closure of cranial sutures, high palate and/or mandibular hypoplasia and/or dental crowding, acroosteolysis of the distal phalanges (hands and/or feet), hypoplastic nails and/or brittle or sparse hair, and at least two of the following skin abnormalities: mottled pigmentation, atrophic skin, sclerodermic skin, or calcified skin nodules. Minor criteria include: lipoatrophy (generalised or partial) of the limbs, joint contractures and shortened phalanges. Major criteria are present in 85–100% of patients reported with MADB, while minor criteria are present in 70–84% of patients reported with MADB [172]. Failure of ossification of the interparietal region of the occipital bone is also a possible pathognomonic sign for MADB [173].

The differential diagnosis between MADA and MADB may be difficult especially in childhood, taking into account that the main clinical features are common [174]. A notable difference between MADA and MADB is the presence of renal disease in the latter group, such as focal segmental glomerulosclerosis and microhematuria [157]. Acanthosis nigricans and other metabolic disturbances have not been reported in MADB [174].

The early diagnosis and slowly progressive nature of MADB offers an opportunity to prevent the complications of the disease, such as renal disease. However, no specific therapeutic options are available. In mice, FTIs and combinations of statins with bisphosphonates have been tested to inhibit farnesylated prelamin A accumulation [131]. Treatment with zoledronate and pravastatin has improved various health indicators and increased median survival from 101 to 179 days [175]. Intravenous pamidronate infusion in a patient with MADB increased bone density but did not prevent osteolysis effectively [160]. Baricitinib alone or combined with FTIs showed beneficial effects on adipogenesis, suggesting potential therapeutic benefits pending further in vivo validation [176]. Lonafarnib corrected nuclear morphology in MADB patient cells, suggesting potential therapeutic benefits despite contradictory findings in some studies [177,178]. Treatment with rapamycin also showed promise in fibroblasts from *ZMPSTE24*-variant carriers, suggesting potential therapeutic avenues [177]. Morpholino antisense oligonucleotides targeting prelamin A reduced senescence markers and improved nuclear abnormalities in MADB patient fibroblasts [179]. Future strategies for premature ageing diseases such as MADB may include gene therapy (CRISPR), cell-based therapies focused on vascular tissue or RNA interference-based therapy [179]. Overall, the optimal therapeutic approach for MADB requires further investigation.

## 8. Nestor-Guillermo Progeria Syndrome

NGPS (#MIM 614008) is a rare autosomal recessive disorder. Genetic studies have identified the causative gene as Barrier-to-Autointegration Factor 1 (*BANF1*), although the reliability of the data remains limited due to the small number of reported cases (only three) [12,180,181]. Recently, another case in heterozygosis has also been reported, showing neuropathy and low weight [182].

Affected individuals appear normal at birth and reach normal developmental milestones. However, by the age of two years, symptoms such as growth retardation and lipodystrophy gradually begin to manifest, although in one case they presented at the age of one year [180]. The syndrome progresses with a marked decrease in mobility affecting both small and large joints over the following years. Hair loss begins during childhood and severe osteolysis becomes evident, mostly affecting the jaw, shoulders and nasal structure. This results in characteristic features such as dry, wrinkled skin and mottled pigmentation, reminiscent of premature ageing. Surprisingly, pubertal development proceeds normally, which distinguishes NGPS from other progeroid syndromes [183].

Individuals with NGPS usually do not present with arteriosclerosis or neurodegeneration until the third and fourth decades of life, an uncommon feature in similar diseases [184]. Despite phenotypic overlaps with HGPS, MAD and APS, NGPS is differentiated by the absence of cardiovascular complications and metabolic abnormalities. Instead, severe osteolysis involving several skeletal structures is a distinguishing feature [12] (Table 2).

NGPS exhibits a chronic progeroid phenotype due to its early onset and slow clinical progression, resulting in relatively prolonged patient survival. The clinical findings of NGPS contrast with those of classic HGPS in several respects, such as less severe growth retardation, increased height, delayed scalp hair loss and persistence of eyebrows and eyelashes [9]. Laboratory investigations have revealed vitamin D2 deficiencies and severe hypoleptinaemia, further characterising the metabolic profile of NGPS [183].

Regarding treatment, synthetic growth hormone was administered to a patient with NGPS at the age of 3 years, despite adequate endogenous growth hormone production. The treatment did not alter the natural progression of the disease [12]. Dental removal and mandibular surgery showed little efficacy in limiting mandibular resorption and may have accelerated bone deterioration [12]. Teriparatide, a recombinant human parathyroid hormone commonly used for the treatment of osteoporosis [185], was the only therapy that reduced bone loss and increased bone mineral density, although its use is not licensed. Unlike bisphosphonates, teriparatide stimulates bone remodelling, which may benefit patients with NGPS sensitive to physical stimulation. However, its long-term use is limited due to concerns about the risk of osteosarcoma [12]. Further research is needed to evaluate the safety and efficacy of aminobisphosphonates and teriparatide in NGPS. Identification of *BANF1* variants associated with progeroid syndrome could lead to new therapeutics [175,186,187] and the development of NGPS mouse models would facilitate the testing of new therapies [12].

## 9. Discussion

The disorders that fall within the family of lipodystrophic laminopathies are defined by the presence of pathogenic variants in the same gene (*LMNA*), and other related genes, in addition to the presence of an impaired adipose tissue pattern. However, there is great clinical heterogeneity among these syndromes, ranging from Dunnigan disease, in which the most relevant trait is precisely adipose tissue dysfunction and lipodystrophy, to the other laminopathies affecting adipose tissue, characterised by the presence of signs of premature ageing (Table 2).

In this sense, the nuclear lamina has attracted the attention of researchers in the field of ageing considering its participation in the maintenance of the nuclear structure, DNA replication, chromatin organisation and gene expression in such a way that variants in genes encoding proteins involved in its structure and/or function can cause these premature ageing syndromes [188]. Thus, progeroid laminopathies have also become excellent sources of information for the understanding of physiological ageing [184] and debate has been opened on the extent to which the mechanisms of progeria and normal ageing may overlap. In fact, it has been speculated that defective prelamin A processing and accumulation may have a role in physiological ageing in relation with decreased expression of ZMPSTE24, although this continues to be a matter of discussion [189].

Although, to date, there is still no clear consensus on what should truly be considered premature ageing, it has recently been proposed that to tag an entity as progeroid at least 40% of ageing symptoms or signs should be present, before the typical age of onset in physiological ageing [180,184]. These ageing symptoms or signs consist not only of a series of external phenotypic features for which there is evidence that these occur as part of physiological ageing in the general population, but also of characteristics involving the internal organs that are likewise important features of ageing (osteoporosis, atherosclerosis, cancer, etc.). Thus, unlike other lipodystrophic laminopathies, although Dunnigan disease does not meet the phenotypic characteristics associated with progeroid syndromes beyond partial lipodystrophy, it is characterised by the development of certain organ abnormalities (such as non-ketotic diabetes mellitus, cardiovascular disease, etc.) at younger ages than for the general population, even leading to a lower average life expectancy [81,190]. This could raise the question of whether Dunnigan disease could be considered not only a monogenic model of lipodystrophy, but also of mild premature senescence. In this sense, whereas in the majority of lipodystrophic laminopathies associated with premature ageing, the accumulation of farnesylated prelamin A is observed [118,153,161], the involvement of prelamin A in the pathogenesis of FPLD2 should be considered with caution. Thus, while there are studies that appear to have shown prelamin A accumulation in peripheral subcutaneous adipose tissue in patients with FPLD2 [14], a more recent study, using an anti-prelamin A monoclonal antibody, did not find such accumulation in cultured fibroblasts from FPLD patients with different variants in the *LMNA* gene [191].

The general group of laminopathies mainly affects mesenchymal tissues (adipose tissue, muscle, bone) [10]. However, this does not occur homogeneously in all syndromes, with different disorders affecting different tissues. The presence of overlapping features is even relatively common [192]. Thus, while in the case of Dunnigan disease, only adipose tissue is affected along with muscle hypertrophy [193,194], in the case of HGPS, MADA, MADB, NGPS and APS, adipose tissue, muscle and bone are affected [195]. In addition, the pattern of lipodystrophy is not the same for each of these diseases. While Dunnigan disease, MADA and some subtypes of APS show partial lipodystrophy, in the case of HGPS, MADB, NGPS and some subtypes of APS the lipodystrophy is generalised [11]. The reason for this variable expressivity in mesenchymal tissue abnormalities, and more specifically in the fat distribution pattern of lipodystrophic laminopathies, is unknown. However, there is recent evidence that changes in the epigenome (such as in the case of genes mediated by HOTAIR or the miR-335 locus, FGF21 or LPL) can affect adipogenesis, conditioning the development and impairment of adipose tissue in a depot-specific manner [21,196]. This could help to understand the adipo-phenotype of the different subtypes of lipodystrophic laminopathies.

On the other hand, in lipodystrophy, the lack of adipocytes prevents the effective storage of lipids, which are, therefore, also accumulated ectopically, mainly in the liver and muscle [197]. This situation, along with a dysfunctional pattern of adipocytokine production, plays a relevant role in the aetopathiogenesis of insulin resistance [63]. Consequently, most lipodystrophy syndromes are characterised by a variable degree of metabolic comorbidities [197]. In addition, taking into account that the severity of insulin resistance and the toxic accumulation of fat is more severe in the generalised forms of lipodystrophy considering that adipose tissue storage capacity is non-existent by definition, patients with classic generalised lipodystrophy are associated with more severe metabolic disease [198]. However, this does not appear to be true for all types of lipodystrophic laminopathies. It is, in fact, striking that in entities in which lipodystrophy is severe, as is the case of NGPS and HGPS, which are characterised by a generalised form of lipodystrophy, the metabolic comorbidities are mild or even absent [12].

There are other multiple broad differences that make a distinction between the different lipodystrophic laminopathies, such as the age at which the first symptoms of the disease appear, ranging from the perinatal stage in the case of MADB to puberty in the case of women with FPLD2, passing through early childhood in the other lipodystrophic laminopathies [193,199]. The differences in lifespan are also striking, even within progeroid syndromes. Thus, while for the rest of the progerias average lifespan is 14 years, in the case of NGPS the patients described presented with a clearly higher life expectancy (>30 years). For this reason, this syndrome is also defined as a chronic progeria, considering the slow clinical course and longer survival [12,181]. In the same way, patients with APS and longer life expectancy (even >50 years) have also been reported [143]. This is consistent with the complex genotype-phenotype associations and clinical heterogeneity of laminopathies [11].

Lipodystrophic laminopathies are considered to be rare disorders, or even ultra-rare disorders based on the prevalence rate of less than 1 per 50,000 individuals defined by the European Union for ultra-rare diseases (https://eur-lex.europa.eu/legal-content/EN/TXT/?uri=CELEX%3A32014R0536&qid=1720375578947, accessed on 7 July 2024). However, it is difficult to speak of prevalence in a rare disease. In fact, for many of these ultra-rare disorders, prevalence cannot be established taking into account the low number of cases described in the literature to date. In addition, while premature ageing syndromes are more clinically recognisable and ultimately more easily diagnosed, in the case of Dunnigan disease the diagnosis is more intricate, especially in men in whom the phenotype is less apparent [41,42]. Furthermore, the clinical heterogeneity previously described even within the same syndrome may also contribute to delays in diagnosis or even misdiagnosis. These factors are of special relevance considering that the initial diagnosis of these diseases is merely clinical. Thus, the estimation of prevalence for these syndromes will not only be overshadowed by the small number of reported cases, but also underestimated due to the late diagnosis or underdiagnosis in some cases. The presence of specialised reference units with multidisciplinary teams for the diagnosis and management of these rare disorders is essential, along with international research cooperation to help carry out studies with larger sample sizes and present a more objective picture of these syndromes.

In conclusion, lipodystrophic laminopathies are ultra-rare disorders due to pathogenic variants in the same gene (*LMNA*), and other related genes, that condition alterations in lamin A processing and, consequently, adipose tissue impairment along with diverse clinical manifestations and comorbidities. Despite the similarities in their aetiopathogenesis, they prove to be individual entities with complex genotype-phenotype associations and great clinical heterogeneity as one of their most fascinating traits. Therefore, there are still many questions to be resolved and, although in recent years new therapies have been developed primarily aimed at the management of their comorbidities, there is still much to do. Thus, international networks should be established in order to better understand these disorders.

## 10. Research Agenda

- There is a need to establish international cooperation and networks through the International Clinical Working Groups of the European Consortium of Lipodystrophies (ECLip) in order to address the real lipodystrophic laminopathies and create registries that bring together relevant information about these ultra-rare pathologies.
- There is also a need to delve deeper into what should really be considered a progeroid syndrome while trying to gather scientific evidence based on consensus among worldwide experts.
- Increasing knowledge of the molecular basis of lipodystrophic laminopathies will make it possible, in the future, to find new specific therapies to shed light on what until now are life-threatening or fatal disorders.
- Attention should be focused on the field of epigenomics and the possibility of modulating clinical expressivity through reversible epigenetic markers. In this sense, epigenetic studies on whole adipose tissue and preadipocytes, including the already-known miR335 but also HOTAIR involvement and other epigenetic marks, could improve understanding of the big phenotypic differences (when comparing FPLD vs. progeroid syndromes), or mild phenotypic differences in FPLD2 pending the specific *LMNA* variants.

## Figures and Tables

**Table 1:** Differential features of Dunnigan disease in comparison with other familial partial lipodystrophy syndromes.

	Dunnigan Disease [30,38,39,40,41,42,43,44,45,46,47,48,49,50,51,52,53,54,55,56,57,58,59,60,61,62,63,64,65,66,67,68,69,70,71,72,73,74,75,76,77,78,79,80,81,82,83,84,85,86,87]	Other FPLD Subtypes [88,89,90,91,92,93,94,95,96,97,98,99,100,101,102,103,104,105,106,107,108,109,110]	
Molecular characteristics	Nuclear lamina alteration Variants in *LMNA* gene - Classic FPLD2: R482W and R482Q variants in exon 8 - Atypical FPLD2: non-codon 482 variants	- Unknown: FPLD1. - Adipogenesis dysregulation: FPLD3 (*PPARG* gene), FPLD9 (*PLAAT3* gene). - Lipid droplet impairment or lipolysis dysregulation: FPLD4 (*PLIN1* gene), FPLD5 (*CIDEC* gene), FPLD6 (*LIPE* gene), FPLD8 (*ADRA2A* gene). - Insulin signal transduction alteration: *AKT2*-related FPLD. - Caveolar function alteration: FPLD7 (*CAV1* gene). - Dysregulation of phosphatidylcholine biosynthesis: *PCYT1A*-related FPLD. - Mitochondrial dysfunction: *MFN2*-related FPLD.	
Type of inheritance	Autosomal dominant/Semi-dominant inheritance	- Polygenic: FPLD1, FPLD7. - Autosomal dominant: FPLD3, FPLD4, FPLD7, FPLD8 and *AKT2*-related FPLD. - Autosomal recessive: FPLD5, FPLD6, FPLD9, *PCYT1A*- and *MFN2*-related FPLD.	
Onset of fat loss	Puberty in women, later in men	- Birth: FPLD7 - Childhood: FPLD1, FPLD4, FPLD5, *PCYT1A*- and *MFN2*-related FPLD - Adolescence: FPLD3, FPLD8 and *MFN2*-related FPLD - Adulthood: FPLD3, FPLD4, FPLD6, *AKT2*- and *MFN2*-related FPLD	
Abnormal fat pattern	- Loss of fat in the limbs, trunk and gluteal region. - Accumulation of fat in the face, neck, chin, axillae, interscapular area and abdominal viscera. - Hypertrophy of mons pubis fat surrounded by subcutaneous lipoatrophy (“Dunnigan sign”). - Subcutaneous lipomas (20%)	- FPLD3: less severe loss of fat. - FPLD6 and *MFN2*-related FPLD: multiple lipomatous masses. - FPLD7: loss of fat in the face and upper body. - FPLD9: adipose tissue loss varies from partial to generalised.	
Adipokine Disturbance	- Leptin levels ranging from low to normal values. - Lower adiponectin levels in comparison with healthy controls	*MFN2*-related FPLD: very low leptin concentrations.	
Clinical features	- Muscular hypertrophy - Myalgias - Phlebomegaly - Hirsutism in women - Acanthosis nigricans and acrochordons	- FPLD1: KöB index > 3.477 - FPLD3: less prominent musculature, phlebomegaly - FPLD6: muscular dystrophy - *PCYT1A*-related FPLD: short stature, muscular atrophy.	
Organ abnormalities and comorbidities	Metabolic abnormalities - Non-ketotic diabetes - Hypertriglyceridaemia, low HDL cholesterol Heart abnormalities - Cardiac hypertrophy - Atrioventricular conduction defects - Heart failure - Early atherosclerosis - Arrhythmias Liver abnormalities - NAFLD - NASH - Cirrhosis Kidney abnormalities - Proteinuria - Chronic renal failure Reproductive abnormalities - Polycystic ovary syndrome - Fertility problems - Miscarriages and stillbirths Others - Hypertension - Acute pancreatitis	- FPLD3: earlier and more severe metabolic complications - FPLD5: diabetes with ketosis. - FPLD6: auto-fluorescent drusen-like retinal deposits. Increased CK levels. - FPLD7: congenital cataracts. - FPLD9: neurological abnormalities (demyelinating neuropathy, intellectual disability). - *MFN2*-related FPLD: peripheral axonal neuropathy.	

FPLD: familial partial lipodystrophy; HDL: high-density lipoprotein; CK: creatine kinase; NAFLD: non-alcoholic fatty liver disease; NASH: non-alcoholic steatohepatitis.

**Table 2:** Differential diagnosis of the main lipodystrophy-associated laminopathies.

Disease	Gene	Inheritance	Dysfunction	Onset of Phenotype	Fat Loss	Clinical Features	Main Comorbidities
FPLD2 or Dunnigan disease [30,38,39,40,41,42,43,44,45,46,47,48,49,50,51,52,53,54,55,56,57,58,59,60,61,62,63,64,65,66,67,68,69,70,71,72,73,74,75,76,77,78,79,80,81,82,83,84,85,86,87] #151660	*LMNA*	AD	Nuclear lamina alteration	Puberty in women, later in men	Partial	Subutaneous lipomas. Muscular hypertrophy, phlebomegaly. “Dunnigan sign”	Diabetes, hypertriglyceridaemia, hepatic steatosis, fertility problems, PCOS, cardiovascular disease, arrhythmias. May associate cardiomyopathy, muscular dystrophy.
HGPS [118,119,120,121,122,123,124,125,126,127,128,129,130,131,132,133,134,135,136,137] #176670	*LMNA*	AD	Nuclear lamina alteration	18–24 months	Generalised	Global alopecia, prominent veins on the scalp, micrognatia, dental crowding.	Bone and joint alterations. Atherosclerosis, cardiovascular disease. Mild metabolic syndrome.
APS [138,139,140,141,142,143,144,145,146,147] -	*LMNA*	AD	Nuclear lamina alteration	Childhood/Early adulthood	Generalised or partial	Phenotypic heterogeneity. Premature greying of hair, alopecia, dental crowding.	Metabolic complications. Valvulopathy, dilated cardiomyopathy. Acroosteolysis and clavicular hypoplasia are absent or mild.
MADA [8,14,159,160,161,162,163,164,165,166,167,168] #248370	*LMNA*	AR	Nuclear lamina alteration	2–4 years	Partial	Growth retardation, beaked nose, mottled skin pigmentation, dental crowding	Bone alterations. Metabolic syndrome. Premature adrenal cortical dysfunction in some cases.
MADB [131,157,169,170,171,172,173,174,175,176,177,178,179] #608612	*ZMPSTE24*	AR	Nuclear lamina alteration	Perinatal	Generalised	Short stature, delayed closure of fontanels, calcified skin nodules.	Musculoskeletal abnormalities. Metabolic syndrome.
NGPS [9,12,180,181,182,183,184,185,186,187] #614008	*BANF1*	AR	Nuclear lamina alteration	2 years	Generalised	Profound skeletal abnormalities.	Bone alterations. No metabolic complications.

FPLD2: familial partial lipodystrophy type 2; HGPS: Hutchinson Gilford progeria syndrome; APS: *LMNA*-atypical progeroid syndrome; MADA: Mandibuloacral dysplasia type A; MADB: Mandibuloacral dysplasia type B; NGPS: Nestor-Guillermo progeria syndrome; AD: autosomal dominant; AR: autosomal recessive; PCOS: polycystic ovary syndrome.

## Data Availability

No new data were analysed in this study. Data sharing is not applicable to this article.
